# Nutrition Care in Cancer Surgery Patients: A Narrative Review of Nutritional Screening and Assessment Methods and Nutritional Considerations

**DOI:** 10.7759/cureus.33094

**Published:** 2022-12-29

**Authors:** Dheeraj Babu Baji, Jay P Patel, Nithin Kumar Konanur Srinivasa, Akshay Gande, Madatala Anusha, Hassaan Dar

**Affiliations:** 1 Research, All India Institute of Medical Sciences, Bhubaneswar, Bhubaneswar, IND; 2 Research, Chirayu Medical College and Hospital, Bhopal, IND; 3 Research, Bangalore Medical College and Research Institute, Bangalore, IND; 4 Research, Gandhi Medical College and Hospital, Secunderabad, IND; 5 Internal Medicine, Sri Devaraj Urs Medical College, Kolar, IND; 6 Research, Jinnah Medical and Dental College, Islamabad, PAK

**Keywords:** nutrition, screening tools, screening of nutritional status, special diet, postoperative nutrition care, preoperative nutrition care, nutritional assesment, onco-surgery

## Abstract

Malignancy is a catabolic state, which is precipitated with surgical intervention. Malnutrition is one of the main risk factors for poor outcomes of cancer surgery. We need to screen oncological patients for malnutrition using standardized screening tools, by which patients found to be at nutritional risk are then referred to a registered dietitian for further management. A detailed assessment is required in such patients, which helps in categorizing the patients based on the severity and rendering proper care. Preoperative nutrition care is often overlooked because of the urgency of operating on a cancer patient. Still, studies have shown preoperative nutritional building gives better surgical outcomes and good postoperative quality of life. Preoperative nutrition care includes both early and late preoperative care. For efficient preoperative nutrition care publishing, standard operating procedures at every healthcare center are recommended. Postoperative nutrition care is given to build the patient tackle the surgical trauma, and their diet mainly includes protein to minimize catabolism. Regardless of the route of nutrition delivery, providing appropriate nutrition care in the postoperative period improves cancer patients' condition drastically. Early postoperative nutrition is studied in different cancer surgeries and is considered ideal in cancer surgical patients. There is a need for consensus on the composition of postoperative nutrition. The diet of a cancer patient should include micronutrients like vitamins D and B and minerals along with the usual nutrition care. The use of special diets like branched-chain amino acids and immune nutrition is to be considered on a case-by-case basis and introducing them into the routine care of a patient needs to be studied extensively.

## Introduction and background

Malignancy induces chronic malnutrition, which is known to have poor postoperative outcomes compared with well-nourished patients undergoing surgery [1-3]. The prevalence of malnutrition in oncological patients ranges from 10% to 85%, depending on the definition of malnutrition, various assessments, and the type of cancer [4,5]. Severe undernutrition is an independent risk factor for increased postoperative morbidity and mortality, prolonged hospital stays (LOS), infections, and high costs in cancer patients [6]. These poor postoperative outcomes give rise to poor quality of life. Efforts to improve the postoperative recovery process primarily focus on intraoperative factors, such as minimally invasive surgery [7], and postoperative interventions like early nutrition and mobilization, designed to ease the return of functional activities and speed up healing [8]. Yet, malnutrition can be said to impact both intraoperative and postoperative outcomes. Perioperative comprehensive nutrition support has not been effectively integrated into the usual care of oncological patients having surgery, and nutritional evaluation in cancer patients requiring surgery is overlooked [9]. Although postponing surgery on a cancer patient is not virtuous, operating on a malnourished patient does not result in a better postoperative prognosis. Therefore, nutrition care in cancer surgery is a real interprofessional challenge [10]. Malnutrition cannot be entirely attributable to disease activity in oncological patients. A definition of malnutrition that takes into account both phenotypical (non-voluntary weight loss, a low body mass index [BMI], and decreased muscle mass) and etiological criteria (reduced food intake or assimilation, inflammation, or disease burden) has been proposed by the International Global Leadership Initiative driven by the clinical nutrition societies [11]. The catabolic state in malignant patients is precipitated by nutrition-impact manifestations (NIM) such as nausea, lack of appetite, dry mouth, early satiety, and diarrhea, which cause the patient to be malnourished, and patients receiving neoadjuvant chemotherapy (NAC) are more likely to experience these symptoms [12]. This undernourished state also negatively affects the immune system, as starvation is accompanied by systemic inflammation, the immune function is further depressed, and there is an accelerated loss of lean body mass [13]. According to a study, there was no difference in infection rates and LOS between individuals with a nutrition risk score of 3 and 4 who received preoperative nutritional therapy and those who did not [14]. This informs us that to provide personalized dietary support for improved outcomes and the optimal use of resources, we need a systematic classification of undernourished oncological patients. Furthermore, we should not treat severely malnourished individuals hastily since doing so might compound the problem and result in refeeding syndrome [15]. Many individual trials and detailed review articles have been published describing various methods of managing a malnourished cancer patient, such as preoperative rehabilitation [16] or postoperative management with fast-track early nutrition of a cancer patient [17], and various studies have investigated different nutrition screening and assessment methods in cancer patients undergoing surgery [18]. However, few succinct reviews on comprehensive nutrition care in cancer surgery integrate nutrition screening into individualized intervention. This review article aimed to look into various aspects and degrees of nutrition care in cancer surgery, emphasizing noteworthy nutritional supplements for specific individuals.

## Review

Malignancy is a public health problem that needs a rigorous course of management. Malnutrition is common in cancer patients and is a concern in treating them. In particular, people undergoing cancer surgery face a difficult recovery from surgery due to their malnourished state.

Nutritional screening and assessment methods

The nutritional care process model (NCPM) is a structured and methodical approach to care that nutrition professionals employ [19]. Before using an NCPM, we need to screen the patients attending the oncology outpatient department or planning for oncological surgery, and these screening tools mentioned in Table 1 are tested by using nutrition assessment tools like subjective global assessment (SGA) [20] and patient-generated subjective global assessment (PG SGA) [21]. A screening tool is considered ideal if it has a high sensitivity; for example, the malnutrition universal screening tool (MUST) has phenotypic criteria such as unintentional weight loss and low BMI and causal criteria such as reduced food intake and illness burden with a sensitivity of 80-86.7% and 96% when executed in mixed cancer types [22] and colorectal cancer surgery, respectively [23]. Nutrition risk screening-2002 (NRS-2002), which applies the same criteria as MUST, has a sensitivity of 67-80% in surgical inpatients and cancer patients [24,25]. Other notable screening techniques with good sensitivity are the abridged patient-generated subjective global assessment (aPG-SGA) and the malnutrition screening test for cancer (MSTC) [26]. 

**Table 1:** Nutrition Risk Screening Tools

Screening tool	Criteria	Intended population	Accuracy of the tool
Malnutrition Universal Screening Tool (MUST)	Phenotypic criteria: unintentional weight loss, low BMI causal criteria: reduced food intake, disease burden	Surgical inpatients, colorectal cancer: preoperative assessment (n = 45) [23]	Sensitivity: 96% Specificity: 75%
		Cancer and non-cancer, surgical inpatients: preoperative evaluation (n = 300) [27]; evaluation performed within 36 hours of admission (n = 120) [25]	Sensitivity: 67.8-85% Specificity: 93-94.4%
Nutritional Risk Screening-2022 (NRS-2002)	Phenotypic criteria: unintentional weight loss, low BMI causal criteria: reduced food intake, disease burden	Oncology outpatients, head and neck cancer, CNS cancer (n = 124) [28]	Sensitivity: 67.5% Specificity: 92.9%
		Surgical inpatients, gastric cancer: evaluation done within 24 hours of admission (n = 80) [24]	Sensitivity: 80% Specificity: 96%
Abridged patient-generated subjective global assessment (aPG-SGA)	Phenotypic criteria: unintentional weight loss, causal criteria: reduced food intake, reduced food assimilation	Oncology outpatients, mixed cancer types (n = 246) [29]; (n = 300) [30]; (n = 90) [31]	Sensitivity: 80.4-96.9% Specificity: 72.3-86.2%
Malnutrition screening test for cancer (MSTC)	Phenotypic criteria: unintentional weight loss, low BMI causal criteria: reduced food intake	Oncology inpatients, mixed cancer types (n = 1057: 800 for formulation, 257 for validation) [26]	Sensitivity: 94% Specificity: 84.2%

Adopting an appropriate screening method depends on the patient's cancer type and the clinical setting, as determined by the global leadership initiative on malnutrition (GLIM) criteria [11]. An attending surgeon or a primary physician can administer these screening methods to rule out malnutrition. Based on the screening tool, patients at risk of malnutrition should be referred to a registered dietitian (RD) for nutritional evaluation. RDs conduct a full dietary evaluation utilizing assessment tools such as SGA and PG-SGA, collecting food and nutrition-related history, anthropometric measurements, biochemical data, health and illness status, social and environmental variables, and functional assessments [32]. Obtaining a nutrition-related history includes dietary habits as well as evaluation of NIM and biochemical assessments such as C-reactive protein (CRP), 25-hydroxyvitamin D [33], and iron levels [34].

These are to be corrected promptly before surgery. Anthropometry includes weight, height, and waist and hip ratio, constituting a comprehensive nutritional assessment [32]. Functional assessments include measuring strength with a hand grip [35] and function with a 6-minute walk test [36]. The most critical aim of nutritional screening and assessment is to produce a diagnosis using generic terminology by describing the reported problem, citing the cause, and presenting evidence of the problem (clinical manifestations). A pre-surgery diagnostic statement might look something like this: "severe chronic malnutrition (problem), related to NIM (etiology), which includes constipation, early satiety, and fatigue (clinical manifestations), as evidenced by meeting 65% of estimated protein requirements, 10% weight loss in the previous six months, and low handgrip strength” [32].

Preoperative nutritional care

When oral food intake is not adequate in providing the required nutrition, especially in cancer patients, alternative ways are to be adopted in providing adequate nutrition. Patients can receive artificial nutrition in three modes: enteral nutrition (EN) (from a tube introduced into the stomach or small intestine); parenteral nutrition (PN) (from an intravenous cannulation, to deliver nutrients to the bloodstream directly); or by a combination of both routes (EN and PN). Surgery is a catabolic insult; enduring this is difficult, particularly for older and malnourished patients having poor functional reserves before surgery [37]. Half of the problem is solved by expressing the diagnosis in standardized language, which enables us to deliver personalized care. One such intervention is NCPM, which is used based on risk stratification. Patients with SGA or PG-SGA (<4) less than four are not malnourished and do not require additional evaluation. They can be given handouts or asked to attend group classes to prevent postoperative malnutrition. Patients with SGA or PG-SGA of 4-8 have moderate or suspected malnutrition and require focused interventions such as nutritional counseling and medical care to alleviate symptoms (NIM). A patient suffering from severe malnutrition (SGA or PG-SGA of 9) should be given specialized care, including oral feeding, EN, and PN [32]. Oncology nurses have a critical role in the nutrition and hydration of cancer patients to improve their survivability and quality of life [38]. In a scoping review on preoperative nutrition in cancer, Brajcich et al. concluded in their study that all cancer patients require prior nutritional counseling, and malnourished patients require a protein-calorie rich diet, immunonutrition (IM) supplementation for gastrointestinal (GI) cancer surgery, and probiotics or synbiotics for colon cancer surgery [1]. In a study on severely malnourished patients who were provided with 7-15 days of preoperative nutrition as total parenteral nutrition (TPN), which led to reduced postoperative complications in comparison with the control group, the authors recommend administering TPN only in severely malnourished patients [39], and an international study by Luca et al. on pancreatic cancer (which has a high mortality rate) surgery patients informed that preoperative nutrition care is vital and it is directly related to the outcome of the surgery and they propose when an artificial nutritional support is needed, EN over PN when possible [40]. A study on gastric cancer patients undergoing gastrectomy with severe nutritional risk (based on the European Society for Clinical Nutrition and Metabolism [ESPEN] definition) found that those who received adequate energy support for at least 10 days had lower surgical site infection than those who did not receive or received inadequate care [41]. ESPEN guidelines recommend 7-14 days of delayed surgery with nutritional replenishment for cancer patients with weight loss of >10% in the past six months, BMI <18.5 kg/m^2^, and albumin <30 g/L or NRS > 5 [10]. Although there is no proper amount of calories recommended for individual cancer types, ESPEN practical guidelines for clinical nutrition in cancer recommend appropriate nutritional support at 25-30 cal/kg/d and 1.5 g/kg/d protein for malnourished during that period [42]. Few studies disagree on preoperative nutrition care, which include a systematic review on preoperative nutrition support in esophageal and gastric cancer patients by Deftereos et al., who posited that there is no strong evidence to determine the ideal diet for nutrition support before upper GI cancer surgery [43], and a retrospective study by Claudino et al. on gastric cancer patients who received IM preoperatively found that there is no reduction in the LOS or postoperative complications and suggested on more studies about benefits of IM [44]. Besides early preoperative nutrition care, late preoperative nutritional support should be provided appropriately. In patients without aspiration issues, liquids are given 2 hours before surgery, and solid food is given 6 hours before surgery as recommended by ESPEN guidelines on clinical nutrition in surgery [10]. Preoperative fasting leads to postoperative insulin resistance and causes preoperative hyperglycemia, which increases the risk of postoperative complications such as increased LOS, renal failure, and infection [45]. To avoid these complications, carbohydrate loading with maltodextrin (50 g packet) mixed in water should be given 3 hours before surgery [46,47]. Furthermore, carbohydrate loading relieves anxiety in the patient [10]. Appropriate use of EN and PN is to be considered, and the ESPEN guideline recommends the decision to be based on the patient's nutrition risk category and previous food intake [10]. They also recommend combining EN and PN if intake within seven days of the start of nutrition supplementation meets less than 50% of the calorie requirement, and PN alone can be given if EN is contraindicated (intestinal obstruction or immediate nutritional building) [10]. A cost analysis study suggested a three-chamber bag to be employed in place of a multi-bottle system in PN as it costs less [48]. Studies on critically ill patients managed with appropriate protocol gave good outcomes [49,50]. So, SOPs for nutrition care and exhibiting them at a healthcare center help in better nutrition care.

Postoperative nutrition care

The principal aim of perioperative nutritional therapy is to provide protein to minimize catabolism while simultaneously maintaining normoglycemia, enough hydration, and avoiding fasting [51]. A study by Bozzetti et al. concluded that postoperative nutritional support in GI cancer regardless of whether an EN, PN, or IM supplementation is administered in the postoperative period has a protective effect with reduced complications in cancer surgeries [52]. Few studies even before the advent of sophisticated intraoperative care indicated that oral nutrition, including oral nutrition supplements (ONS) and/or a hospital-balanced meal, can be delivered immediately after surgery [53,54]; nonetheless, ESPEN guidelines recommend that oral nutrition be administered as tolerated [10]. Obermair et al. introduced early oral and EN intake was found to be safer in gynecological surgeries, with a shorter LOS and intestinal recovery time and fewer complications [55]. A Cochrane review on providing EN within 24 hours of lower GI surgery established that it helped in faster recovery with fewer complications [56], and this is corroborated by Lassen et al. in major upper GI procedures such as gastrectomy or Whipple's procedure providing feeding at the patients’ will [57]. Early normal food or EN, including clear liquids on the first or second postoperative day, does not compromise anastomosis healing in the colon and rectum [54,57,58], has a lower LOS [59], and does not increase mortality [60]. In colon cancer surgery patients, postoperative nutrition on day 1 is an independent prognostic factor of five-year survival and mortality [61,62]. Early EN has been shown to improve outcomes in patients with total gastrectomy [63] or minimally invasive esophagectomy [64] for respective cancers. Feeding jejunostomy may be superior to nasojejunal or duodenal tubes in delivering EN due to unintentional dislodgement of the latter in a patient who underwent major upper GI surgery such as esophagectomy or pancreatic surgery [10,65]. Even in gynecological cancer surgery, numerous randomized studies on early meal consumption have been chosen. Most studies claimed to provide oral caloric intake within 24 hours following surgery [66,67]. A study on elective cancer surgery by Fachini et al. about resuming food intake after 24 hours post-surgery found an increased likelihood of a longer ICU stay and a greater infection rate in cancer patients [68]. There is a dearth of controlled evidence about using EN and PN (dual nutrition after elective surgery); however, our goal is to increase calorie intake [10]. Furthermore, there is no consensus on the composition of a postoperative diet; nonetheless, a high-protein diet may reduce the incidence of complications [61]. Despite substantial data supporting early postoperative nutrition support, it is not done due to patient rejection due to anxiety, appetite loss, and other causes [69]. However, it has been ascertained that patients who receive nutrition counseling on the second day of post-GI surgery improve their dietary intake [70].

Special nutrition care considerations

The regular nutrition care described above for malnourished patients tends to focus on increased caloric intake and adequate protein supplementation, which is sometimes not optimal. This review also highlights a few essential recommendations for implementing particular evidence-based dietary ramifications. Markotic et al. concluded in a study on colorectal cancer patients that 25-hydroxy vitamin D (25 (OH) D) levels may have prognostic value and are strongly affected by surgery (a decrease in serum 25 (OH) D levels), as well as confirmed that higher postoperative 25 (OH) D levels were associated with improved survival in colorectal cancer patients [71]. Before and after surgery, 25 (OH) D levels are typically low in colorectal cancer patients [72]. Low 25 (OH) D levels are also observed in advanced T or N-stage breast cancer, but patients with normal 25 (OH) D levels have excellent one-year survival [73]. In breast cancer patients who have received NAC, vitamin D insufficiency is a barrier to achieving a pathological complete response [74]. At the time of cancer diagnosis, 70-80% of breast cancer patients have 25 (OH) D levels below the standard lower limit, which leaps during NAC [75]. However, the cause of its deficiency is unknown; it is usually attributed to the function of 25 (OH) D in the process of tumor genesis in cancer [74]. Vitamin B supplementation is necessary for cancer patients. As fatigue is a significant problem for postoperative patients, Vitamin B12 and folate supplements are typically used to treat chronic fatigue syndrome [76] and can be safely supplemented in cancer patients. Similarly, hyperhomocysteinemia linked to postoperative cognitive impairment in surgical oncology patients can be treated with the same [77]. Circulating pyridoxal-5'-phosphate (PLP) levels (an active substrate of vitamin B6) are known to be low in inflammatory conditions [78]. Koole et al. discovered that vitamin B6 supplementation in colorectal cancer patients improved their quality of life with less fatigue, as evidenced by optimal PLP levels in those patients [79].

Elderly patients with colorectal cancer have micronutrient deficiencies or consume vitamins and minerals in lower than recommended amounts before and after surgery; 50% of the required multivitamin dose can be provided orally [80]. Humans need the branched-chain amino acids (BCAAs) leucine, isoleucine, and valine [81]. They are protein synthesis substrates and protein turnover regulators [82]. A decrease in muscle protein synthesis and reduced glutamine availability due to cancer contribute to an impaired immune response in those patients, which is worsened by surgical stress [83]. In addition, patients who utilized BCAAs throughout the preoperative and postoperative phases had a 38% decreased infection incidence and a low ascites rate [84]. However, based on a limited number of clinical trials, BCAAs cannot be recommended routinely and require additional investigation through extensive multicenter studies. Calorie restriction or intermittent fasting (IF) may seem like an oxymoron in a cancer patient, but when implemented correctly, IF can cause a decrease in IGF-1 and insulin levels, which enhances cancer cells' sensitization to chemotherapy, such as NAC, while also promoting hematopoietic stem cell-based regeneration and reversing immunosuppressive action [85]. Numerous human studies on the role of IF in postoperative recurrence are still ongoing [86]. It is worth noting, the Mediterranean diet improves the quality of life for breast cancer patients [87]. Probiotics are given to patients undergoing surgery for GI cancer because they stimulate the immune system and have anti-inflammatory properties [88]. IM is a relatively new diet consisting of arginine, omega-3 fatty acids, and ribonucleotides that are used during the perioperative period to reduce morbidity, mortality, and LOS [89], primarily in malnourished patients [90], and is superior to ONS and no supplements [91]. However, due to the high cost of IM, it cannot be recommended as standard nutrition care for oncological surgery patients.

As a whole, there is compelling evidence evaluating nutrition screening and assessment, preoperative and postoperative nutrition care, and special nutrition considerations, with numerous randomized controlled trials throughout the extensive medical literature. However, a few limitations must be taken into consideration when interpreting the findings of the current review. The disease populations in the studies included in this review are diverse. Many studies excluded high-risk patients, and in some studies, the outcomes were dependent on other interventions along with nutrition care. Second, the focus of this review was much more on nutritional screening and assessment, and it did not go into great detail about the composition of nutrition to be delivered.

## Conclusions

Severe malnutrition is an independent risk factor for increased postoperative morbidity in cancer surgery patients. Personalized nutrition care (based on nutrition risk category and clinical manifestations) will lead to better outcomes in cancer surgery. Perioperative comprehensive nutrition support has not been effectively integrated into the usual care of oncological patients having surgery, particularly nutritional evaluation is overlooked in those patients. Nutrition care should be personalized based on nutritional risk using assessment tools. An attending surgeon or primary physician can screen and refer the patient to an RD if the patient is found to be at risk of malnutrition to provide a diagnosis in generic terminology. A certain mode of nutrition delivery will be decided based on the patient's nutrition risk category. Therefore, there is a need for more randomized trials to establish appropriate nutrition for the malnourished in individual cancer types. SOP for nutrition care must be published in every healthcare center. Patients who received nutrition counselling improved their dietary intake. Apart from providing a high-calorie-protein diet, the nutrition should include micronutrient supplementation, especially vitamins D and B and minerals. Calorie restriction may have a role in cancer, currently under investigation. Other special supplements are recommended on a case-by-case basis. We recommend further studies on the composition of the postoperative diet in individual cancer types and special dietary requirements to be used as nutritional support in malignancy patients undergoing surgery.
